# Comparison of new implantation of cardiac implantable electronic device between tertiary and non-tertiary hospitals: a Korean nationwide study

**DOI:** 10.1038/s41598-021-83160-w

**Published:** 2021-03-15

**Authors:** Seungbong Han, Gyung-Min Park, Yong-Giun Kim, Ki Won Hwang, Chang Hee Kwon, Jae-Hyung Roh, Sangwoo Park, Ki-Bum Won, Soe Hee Ann, Shin-Jae Kim, Sang-Gon Lee

**Affiliations:** 1 Department of Biostatistics, College of Medicine, Korea University, Seoul, Korea; 2 Department of Cardiology, Ulsan University Hospital, University of Ulsan College of Medicine, Ulsan, Korea; 3 Division of Cardiology, Pusan National University Yangsan Hospital, Pusan National University of Medicine, Yangsan, Korea; 4 Division of Cardiology, Department of Internal Medicine, Konkuk University Medical Center, Konkuk University School of Medicine, Seoul, Korea; 5 Division of Cardiology, Chungnam National University Sejong Hospital, Chungnam National University School of Medicine, Sejong, Korea

**Keywords:** Cardiac device therapy, Arrhythmias, Outcomes research

## Abstract

This study compared the characteristics and mortality of new implantation of cardiac implantable electronic device (CIED) between tertiary and non-tertiary hospitals. From national health insurance claims data in Korea, 17,655 patients, who underwent first and new implantation of CIED between 2013 and 2017, were enrolled. Patients were categorized into the tertiary hospital group (n = 11,560) and non-tertiary hospital group (n = 6095). Clinical outcomes including in-hospital death and all-cause death were compared between the two groups using propensity-score matched analysis. Patients in non-tertiary hospitals were older and had more comorbidities than those in tertiary hospitals. The study population had a mean follow-up of 2.1 ± 1.2 years. In the propensity-score matched permanent pacemaker group (n = 5076 pairs), the incidence of in-hospital death (odds ratio [OR]: 0.76, 95% confidence interval [CI]: 0.43–1.32, *p* = 0.33) and all-cause death (hazard ratio [HR]: 0.92, 95% CI 0.81–1.05, *p* = 0.24) were not significantly different between tertiary and non-tertiary hospitals. These findings were consistently observed in the propensity-score matched implantable cardioverter-defibrillator group (n = 992 pairs, OR for in-hospital death: 1.76, 95% CI 0.51–6.02, *p* = 0.37; HR for all-cause death: 0.95, 95% CI 0.72–1.24, *p* = 0.70). In patients undergoing first and new implantation of CIED in Korea, mortality was not different between tertiary and non-tertiary hospitals.

## Introduction

The use of cardiac implantable electronic devices (CIEDs), including permanent pacemaker (PPM), cardiac resynchronization therapy with pacemaker (CRT-P) or defibrillator (CRT-D), and implantable cardioverter-defibrillator (ICD), has been increasing in the Western^1–3^ and Asian countries^4^.

In South Korea, health care services are provided through primary and secondary care facility. While primary care services are provided through non-tertiary hospital (clinics, hospitals, and general hospitals), patients can access secondary care through tertiary hospitals (advanced general hospitals)^5^. The health care delivery system is introduced to utilize medical resources efficiently (to limit patients’ herd behavior to seek tertiary hospital services), establish the roles of medical institutions, and help curb the rise in national medical expenditures and to secure financial sustainability^6^. However, patients themselves can choose their medical providers, giving them access to medical institutions without too many restrictions. With no strict gatekeeping system, it is relatively easy for patients to access secondary care in tertiary hospitals. In addition, patients generally prefer tertiary hospitals to non-tertiary hospitals because they believe that the quality of care that is provided by tertiary hospitals is better than that of non-tertiary hospitals^7^. These engender the concentration of patients in tertiary hospitals and results in the waste of health care resource and delay of timely treatment^8^. This tendency is also observed in CIED procedures. Although the number of tertiary hospitals was only about a quarter of total number of medical institutions that performed pacemaker procedure, more than 60% of pacemaker procedures were performed in tertiary hospitals^9^. However, there is paucity of data comparing the clinical outcomes of CIED procedures between tertiary hospitals and non-tertiary hospitals. Therefore, we aimed to compare the characteristics and mortality of new implantation of CIED between tertiary hospitals and non-tertiary hospitals.

## Methods

### Data sources

In South Korea, all healthcare providers had to join the national health insurance (NHI) system on a fee-for-service basis. The Health Insurance Review & Assessment Service (HIRA) is a quasi-governmental organization that systematically reviews medical fees to minimize the risk of redundant and unnecessary medical services. Thus, all NHI claims are reviewed by the HIRA^10^. For this study, data from 2013 to 2017 claims records of the HIRA were used. Patient information was anonymized and de-identified in the claims database of the HIRA. Diagnosis codes were used according to the International Classification of Diseases, 10th Revision (ICD-10). In addition, specific information about the procedure, devices, and drugs were identified by codes from the HIRA database^10^. This study was approved by the Institutional Review Board at Ulsan University Hospital, Ulsan, Korea. The requirement for informed consent was waived by the Institutional Review Board at Ulsan University Hospital because of the anonymity of the patients and the nonintrusive nature of the study. All methods were performed in accordance with the relevant guidelines and regulations.

### Study population

From the claims database of the HIRA between July 2013 and June 2017, we identified patients who had undergone implantation of CIED (such as PPM, CRT-P, ICD, and CRT-D) and their corresponding device codes. To examine patients with first and new implantation of CIED, we excluded patients with generator change, generator removal, upgrade of CIED, and revision of leads. We also excluded patients with insufficient information of CIED type (n = 36) in the HIRA database. To identify the comorbid condition of patients, we selected those with at least 6 months of information prior to the index day. Furthermore, patients were categorized as PPM group (including PPM or CRT-P) and ICD group (including single/dual chamber ICD or CRT-D).

Then, patients were classified into the tertiary hospital group and non-tertiary hospital group according to the hospital where CIED procedures were performed. In the present study, the definition of tertiary hospitals is advanced general hospitals that were selected and authorized by the Ministry of Health and Welfare of South Korea during the study period (2013–2017)^11^.

### Study variables

The ICD-10 codes were used to identify comorbid conditions such as diabetes, diabetes with chronic complications, dyslipidemia, hypertension, congestive heart failure, peripheral vascular disease, cerebrovascular disease, chronic pulmonary disease, moderate to severe liver disease, and renal disease. The codes also identified cancer, rheumatic disease, atrial fibrillation, ventricular tachyarrhythmia (ventricular tachycardia [ICD-10 codes I47.0, and I47.2], ventricular fibrillation or flutter [ICD-10 codes I49.0]), and aborted cardiac arrest (ICD-10 codes I46.X) within 6 months prior to the index day^10,12,13^. The Charlson comorbidity index was obtained from the ICD-10 codes^10^. In the HIRA database, all prescribed medications were recorded with rigorous accuracy. Patients were considered to have hypertension, diabetes, and dyslipidemia if anti-hypertensive, anti-diabetic, and anti-dyslipidemic drugs were identified from the medication codes within 6 months prior to the index day^10,14^. For ICD implantation, ventricular tachyarrhythmia and aborted cardiac arrest within 6 months prior to the index day or at the index hospitalization were classified as indications for secondary prevention and the rest were classified as indications for primary prevention.

### Clinical outcomes

All-cause death was identified by all in- and out-patient claims that indicated death. In-hospital death was defined as death occurred during the index hospitalization. In this study, for the evaluation of clinical outcomes, the HIRA database was used until December 2017.

### Statistical analysis

All baseline patient characteristics and comorbid conditions were summarized as mean ± standard deviation or frequency (percentage) for continuous or categorical variables, respectively. We evaluated whether there are differences for in-hospital mortality and all-cause death rates between tertiary and non-tertiary hospital group. We conducted separate analyses of the PPM and ICD groups. We also conducted subgroup analyses for ICD group according to subtypes of ICD (single/dual chamber ICD and CRT-D). Baseline patient characteristics were compared between tertiary and non-tertiary hospital group using the two sample T-test or the Fisher’s exact test. For the in-hospital mortality, we used the logistic regression model, while we used the Cox proportional hazards regression model for the all-cause mortality rate analysis. In all regression analyses, the reference was the non-tertiary hospital group. We employed the propensity-score matching analysis to reduce the impact of potential confounding effects on the mortality risk comparison. The propensity-scores were derived nonparametrically using the variables of age, gender, hypertension, diabetes, diabetes with chronic complications, dyslipidemia, congestive heart failure, peripheral vascular disease, cerebrovascular disease, chronic pulmonary disease, moderate to severe liver disease, renal disease, cancer, rheumatic disease, atrial fibrillation, ventricular tachyarrhythmia, aborted cardiac arrest, Charlson comorbidity index, type of CIED, and indication of ICD. We used the nearest neighbor matching approach with a caliper size of 0.2 and evaluated the matching quality by measuring the covariate balancing in the matched set. We computed standardized differences in means between the two groups and considered the covariate balance achieved as long as the absolute standardized difference is less than 0.2. All standardized differences in the covariates were less than 0.05. To account for the matched pairs, we used the generalized estimating equations for the in-hospital mortality, as well as the Cox regression model with the sandwich standard errors for time to event outcome of the all-cause mortality. All data analyses were performed using the R software version 3.6.3 (R Foundation for Statistical Computing, Vienna, Austria; www.r-project.org). R ‘MatchIt’ package was used for the propensity-score matching. A *p*-value < 0.05 was considered significant for all two-sided tests.

## Results

### Study population and baseline characteristics

**Figure 1:**
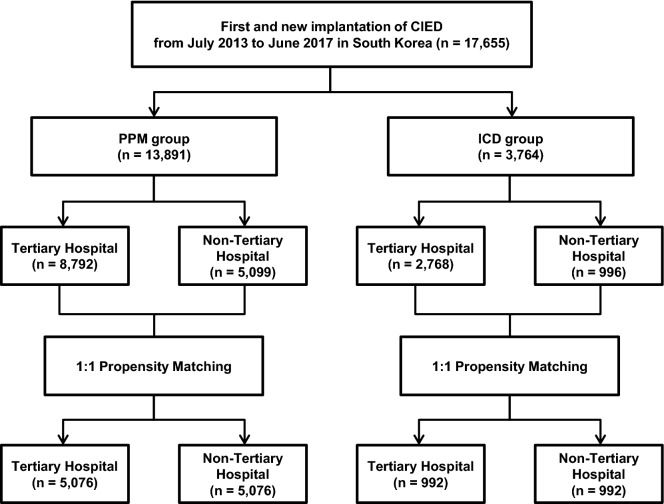
Diagrammatic representation of the study. CIED, cardiac implantable electronic device; ICD, implantable cardioverter-defibrillator; PPM, permanent pacemaker.

Between July 2013 and June 2017, a total of 17,655 patients undergoing first and new implantation of CIED were identified from the claims database of HIRA (Fig. 1). The mean age of study participants was 68.5 ± 13.6 years and 8596 (48.7%) were male. Hypertension, diabetes, dyslipidemia, congestive heart failure, and atrial fibrillation were observed in 13,311 (75.4%), 6310 (35.7%), 10,268 (58.2%), 5232 (29.6%), and 4147 (23.5%) patients, respectively. The annual number of CIED procedures was had significantly increased during the study period (Table 1). According to the hospital categories, 11,560 (65.5%) patients were in the tertiary hospital group and 6095 (34.5%) patients were in the non-tertiary hospital group. The number of hospitals providing CIED (including PPM, CRT-P, ICD, or CRT-D) in the tertiary hospital group and non-tertiary hospital group were 47 and 127, respectively. Tertiary hospital group performed more CIED procedures (61.9 ± 53.4 per year) than non-tertiary hospital group (11.9 ± 19.0 per year).

**Table 1:** Characteristics of patients undergoing new implantation of cardiac implantable electronic device and of hospitals that performed new implantation of cardiac implantable electronic device in tertiary and non-tertiary hospital between 2013 and 2017.

Characteristics	PPM group (n = 13,891)			ICD group (n = 3764)		
	Tertiary hospital	Non-tertiary hospital	*p* value	Tertiary hospital	Non-tertiary hospital	*p* value
	(n = 8792)	(n = 5099)		(n = 2768)	(n = 996)	
Enrolled number (%)			0.012			0.016
July 2013 to June 2014	1970 (22.4%)	1051 (20.6%)		553 (20.0%)	180 (18.1%)	
July 2014 to June 2015	2096 (23.8%)	1168 (22.9%)		696 (25.1%)	213 (21.4%)	
July 2015 to June 2016	2309 (26.3%)	1382 (27.1%)		729 (26.3%)	279 (28.0%)	
July 2016 to June 2017	2417 (27.5%)	1498 (29.4%)		790 (28.5%)	324 (32.5%)	
Age, years	70.5 ± 12.0	72.9 ± 10.7	< 0.001	57.1 ± 15.4	60.5 ± 14.6	< 0.001
CIED rate by age category			< 0.001			< 0.001
< 20	18 (0.2%)	5 (0.1%)		37 (1.3%)	3 (0.3%)	
20–60	1581 (18.0%)	653 (12.8%)		1477 (53.4%)	442 (44.4%)	
61–80	5544 (63.1%)	3161 (62.0%)		1170 (42.3%)	500 (50.2%)	
> 80	1648 (18.7%)	1280 (25.1%)		84 (3.0%)	51 (5.1%)	
Male (%)	3723 (42.3%)	2158 (42.3%)	0.99	1990 (71.9%)	725 (72.8%)	0.62
Comorbid conditions (%)						
Hypertension	6538 (74.4%)	4052 (79.5%)	< 0.001	1968 (71.1%)	753 (75.6%)	0.006
Diabetes	2971 (33.8%)	1929 (37.8%)	< 0.001	981 (35.4%)	405 (40.7%)	0.004
Diabetes with chronic complications*	8 (0.1%)	12 (0.2%)	0.04	2 (0.1%)	2 (0.2%)	0.29
Dyslipidemia	4971 (56.5%)	3066 (60.1%)	< 0.001	1585 (57.3%)	646 (64.9%)	< 0.001
Congestive heart failure	1894 (21.5%)	1218 (23.9%)	0.002	1521 (54.9%)	599 (60.1%)	0.005
Peripheral vascular disease	1236 (14.1%)	807 (15.8%)	0.005	221 (8.0%)	77 (7.7%)	0.84
Cerebrovascular disease	1877 (21.3%)	1218 (23.9%)	0.001	319 (11.5%)	131 (13.2%)	0.19
Chronic pulmonary disease	1815 (20.6%)	1058 (20.7%)	0.90	538 (19.4%)	225 (22.6%)	0.04
Moderate to severe liver disease	21 (0.2%)	12 (0.2%)	> 0.99	0 (0.0%)	0 (0.0%)	N/A
Renal disease	660 (7.5%)	388 (7.6%)	0.84	259 (9.4%)	94 (9.4%)	0.95
Cancer	303 (3.4%)	137 (2.7%)	0.01	57 (2.1%)	22 (2.2%)	0.80
Rheumatic disease	9 (0.1%)	11 (0.2%)	0.11	12 (0.4%)	2 (0.2%)	0.38
Atrial fibrillation	2273 (25.9%)	1091 (21.4%)	< 0.001	578 (20.9%)	205 (20.6%)	0.86
Ventricular tachyarrhythmia	126 (1.4%)	50 (1.0%)	0.02	537 (19.4%)	145 (14.6%)	0.001
Aborted cardiac arrest	18 (0.2%)	16 (0.3%)	0.22	446 (16.1%)	113 (11.3%)	< 0.001
Charlson comorbidity index	1.83 ± 1.63	1.94 ± 1.63	< 0.001	1.89 ± 1.62	2.12 ± 1.77	0.001
Procedure^§^ volume of hospital per year	61.9 ± 53.4	11.9 ± 19.0	< 0.001	61.9 ± 53.4	11.9 ± 19.0	< 0.001
Type of CIED						
PPM						
Single chamber	1290 (14.7%)	930 (18.2%)	< 0.001			
Dual chamber	7404 (84.2%)	4146 (81.3%)	< 0.001			
CRT–P	22 (0.3%)	12 (0.2%)	> 0.99			
Epicardial system	76 (0.9%)	11 (0.2%)	< 0.001			
ICD						
Single chamber				1305 (47.1%)	527 (52.9%)	0.002
Dual chamber				935 (33.8%)	276 (27.7%)	< 0.001
CRT–D				528 (19.1%)	193 (19.4%)	0.85
Indication of ICD	–	–	–	–		< 0.001
Primary prevention				1073 (38.8%)	451 (45.3%)	
Secondary prevention	–	–	–	1695 (61.2%)	545 (54.7%)	
In-hospital mortality	34 (0.4%)	29 (0.6%)	0.15	16 (0.6%)	4 (0.4%)	0.62

Data are reported as mean ± SD or as number (%).

CIED, cardiac implantable electronic device; CRT–D, cardiac resynchronization therapy with defibrillator; CRT–P, cardiac resynchronization therapy with pacemaker; ICD, implantable cardioverter-defibrillator; PPM, permanent pacemaker.

*Including diabetic nephropathy, retinopathy, or neuropathy.

^§^First and new implantation of CIED including PPM, CRT-P, ICD, and CRT-D.

### Tertiary hospital versus non-tertiary hospital in PPM group

According to the CIED procedures, the study participants who underwent PPM or CRT-P implantation were classified as PPM group (n = 13,891). Among them, patients were categorized into the tertiary hospital group (n = 8792) and non-tertiary hospital group (n = 5099). Patients in non-tertiary hospital group were older and had more comorbidities than those in tertiary hospital group (Table 1). Tertiary hospital group performed more implantation of dual chamber and epicardial system pacemaker compare to non-tertiary hospital group.

**Figure 2:**
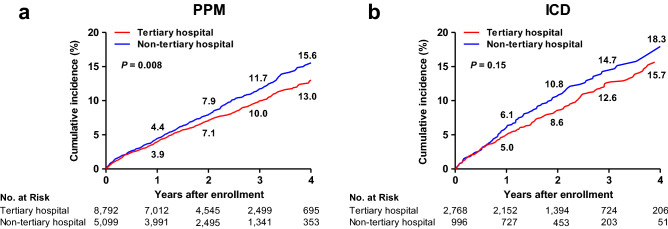
Unadjusted cumulative incidence rates for all-cause deaths in the study population. Cumulative incidence curves are shown for all-cause death in patients with PPM group (**a**) and in those with ICD group (**b**). The numbers in each figure represent the cumulative incidence rates at each time point. All *p* values were calculated with the use of the log-rank test. ICD, implantable cardioverter-defibrillator; PPM, permanent pacemaker.

In-hospital mortality of tertiary and non-tertiary hospital group was 0.4% and 0.6%, respectively. There was no significant difference in the in-hospital mortality between tertiary and non-tertiary hospital group (unadjusted odds ratio [OR] of tertiary hospital: 0.68, 95% confidence interval [CI]: 0.41–1.12, *p* = 0.13). During a mean follow-up of 2.2 ± 1.2 years, non-tertiary hospital group was more likely to have all-cause death than tertiary hospital group (unadjusted hazard ratio [HR] of tertiary hospital: 0.85, 95% CI 0.76–0.96, *p* = 0.008). Figure 2a shows the unadjusted cumulative incidence rates for all-cause deaths of the two groups.

After propensity-score matching, there were 5076 matched pairs. In the matched cohort, there were no other significant differences between tertiary and non-tertiary hospital group for any of the covariates (Table 2). There was no significant difference in terms of the incidence of in-hospital mortality between tertiary and non-tertiary hospital group (OR of tertiary hospital: 0.76, 95% CI 0.43–1.32, *p* = 0.33). During the follow-up period (mean, 2.1 ± 1.2 years), there was no difference in the incidence of all-cause death (HR of tertiary hospital: 0.92, 95% CI 0.81–1.05, *p* = 0.24) between tertiary and non-tertiary hospital group (Table 3).

**Table 2:** Baseline characteristics of the propensity-score matched patients undergoing new implantation of cardiac implantable electronic device in tertiary and non-tertiary hospital.

Characteristics	PPM group (n = 5076 pairs)			ICD group (n = 992 pairs)		
	Tertiary hospital	Non-tertiary hospital	*p* value	Tertiary hospital	Non-tertiary hospital	*p* value
	(n = 5076)	(n = 5076)		(n = 992)	(n = 992)	
Age, years	72.8 ± 10.8	72.9 ± 10.7	0.90	60.2 ± 14.0	60.5 ± 14.6	0.16
Male (%)	2161 (42.6%)	2151 (42.4%)	0.95	729 (73.5%)	722 (72.8%)	0.62
Comorbid conditions (%)						
Hypertension	4017 (79.1%)	4030 (79.4%)	0.18	740 (74.6%)	750 (75.6%)	0.26
Diabetes	1904 (37.5%)	1914 (37.7%)	0.82	408 (41.1%)	402 (40.5%)	0.81
Diabetes with chronic complications*	7 (0.1%)	8 (0.2%)	0.07	1 (0.1%)	2 (0.2%)	> 0.99
Dyslipidemia	3009 (59.3%)	3045 (60.0%)	0.98	627 (63.2%)	643 (64.8%)	0.80
Congestive heart failure	1191 (23.5%)	1205 (23.7%)	0.65	578 (58.3%)	596 (60.1%)	0.47
Peripheral vascular disease	787 (15.5%)	798 (15.7%)	0.54	69 (7.0%)	77 (7.8%)	0.07
Cerebrovascular disease	1200 (23.6%)	1206 (23.8%)	0.47	125 (12.6%)	131 (13.2%)	0.53
Chronic pulmonary disease	1020 (20.1%)	1054 (20.8%)	0.90	225 (22.7%)	224 (22.6%)	0.83
Moderate to severe liver disease	14 (0.3%)	11 (0.2%)	0.69	0 (0.0%)	0 (0.0%)	N/A
Renal disease	394 (7.8%)	386 (7.6%)	0.32	85 (8.6%)	94 (9.5%)	0.87
Cancer	132 (2.6%)	137 (2.7%)	0.21	16 (1.6%)	18 (1.8%)	0.39
Rheumatic disease	9 (0.2%)	9 (0.2%)	0.45	1 (0.1%)	2 (0.2%)	> 0.99
Atrial fibrillation	1128 (22.2%)	1091 (21.5%)	0.77	192 (19.4%)	205 (20.7%)	0.44
Ventricular tachyarrhythmia	59 (1.2%)	50 (1.0%)	0.56	139 (14.0%)	145 (14.6%)	0.73
Aborted cardiac arrest	15 (0.3%)	16 (0.3%)	0.72	114 (11.5%)	112 (11.3%)	0.59
Charlson comorbidity index	1.91 ± 1.62	1.93 ± 1.62	0.59	2.05 ± 1.63	2.10 ± 1.72	0.71
Type of CIED						
PPM			0.40			
Single chamber	897 (17.7%)	913 (18.0%)		–	–	
Dual chamber	4148 (81.7%)	4140 (81.6%)		–	–	
Epicardial system, CRT–P	31 (0.6%)	23 (0.5%)		–	–	
ICD						0.59
Single chamber	–	–		508 (51.2%)	525 (52.9%)	
Dual chamber	–	–		276 (27.8%)	275 (27.7%)	
CRT–D	–	–		208 (21.0%)	192 (19.4%)	
Indication of ICD	–	–				0.41
Primary prevention				444 (44.8%)	448 (45.2%)	
Secondary prevention	–	–		548 (55.2%)	544 (54.8%)	

Data are reported as mean ± SD or as number (%).

CIED, cardiac implantable electronic device; CRT–D, cardiac resynchronization therapy with defibrillator; CRT–P, cardiac resynchronization therapy with pacemaker; ICD, implantable cardioverter-defibrillator; PPM, permanent pacemaker.

*Including diabetic nephropathy, retinopathy, or neuropathy.

**Table 3:** Propensity score matched in-hospital mortality and all-cause death of patients undergoing new implantation of cardiac implantable electronic device in tertiary and non-tertiary hospital.

Propensity-score matched analysis	PPM group (n = 5076 pairs)		ICD group (n = 992 pairs)	
	Tertiary hospital compared to non-tertiary hospital			
	Odds ratio (95% CI)	*p* value	Odds ratio (95% CI)	*p* value
In-hospital mortality	0.76 (0.43–1.32)	0.33	1.76 (0.51–6.02)	0.37
	Hazard ratio (95% CI)	*p* value	Hazard ratio (95% CI)	*p* value
All-cause death	0.92 (0.81–1.05)	0.24	0.95 (0.72–1.24)	0.70

CI, confidence interval; ICD, implantable cardioverter-defibrillator; PPM, permanent pacemaker.

### Tertiary hospital versus non-tertiary hospital in ICD group

We also analyzed the patients with ICD group including single/dual chamber ICD or CRT-D (n = 3764). Among them, patients were categorized into the tertiary hospital group (n = 2768) and non-tertiary hospital group (n = 996). Patients in non-tertiary hospital group were older and had more comorbidities than those in tertiary hospital group (Table 1). Tertiary hospital group performed more implantation of dual chamber ICD compare to non-tertiary hospital group. In addition, tertiary hospital group performed more implantation of ICD for secondary prevention compare to non-tertiary hospital group.

In-hospital mortality of tertiary and non-tertiary hospital group was 0.6% and 0.4%, respectively. There was no significant difference in the in-hospital mortality between tertiary and non-tertiary hospital group (unadjusted OR of tertiary hospital: 1.44, 95% CI 0.48–4.32, *p* = 0.51). During a mean follow-up of 2.1 ± 1.2 years, there was no significant difference in all-cause death between the two groups (unadjusted HR of tertiary hospital: 0.85, 95% CI 0.67–1.06, *p* = 0.15). Figure 2b shows the unadjusted cumulative incidence rates for all-cause deaths of the two groups.

After propensity-score matching, there were 992 matched pairs. In the matched cohort, no significant differences were observed in terms of covariates between the two groups (Table 2). There was no significant difference in terms of the incidence of in-hospital mortality between the two groups (OR of tertiary hospital: 1.76, 95% CI 0.51–6.02, *p* = 0.37). In addition, the incidence of all-cause death (HR of tertiary hospital: 0.95, 95% CI 0.72–1.24, *p* = 0.70) was not different between tertiary and non-tertiary hospital group during the follow-up period (mean, 2.0 ± 1.2 years) (Table 3).

We also conducted subgroup analyses for ICD group according to subtypes of ICD (single/dual chamber ICD and CRT-D). Baseline characteristics of both ICD subtypes were shown in Supplementary Table S1 online. After propensity-score matching, there were no significant differences between tertiary and non-tertiary hospital group for any of the covariates in both ICD subtypes (Supplementary Table S2 online). In both subtypes of ICD, there were no significant differences in the incidence of in-hospital mortality and all-cause death between tertiary and non-tertiary hospital group (Supplementary Table S3 online).

## Discussion

This study aimed to compare the characteristics and mortality of first and new implantation of CIED between tertiary and non-tertiary hospitals. The major findings of the present study using NHI claims data in South Korea are as follows: (1) patients in non-tertiary hospital group were older and had more comorbidities than those in tertiary hospital group; (2) after propensity-score matching, the incidences of in-hospital death and all-cause death were not significantly different between tertiary and non-tertiary hospitals in PPM and ICD group.

In this study, patients in non-tertiary hospitals were older and had more comorbidities compare to those in tertiary hospitals. This finding could be explained by the location of the tertiary (mostly urban region) and non-tertiary (mostly suburban and rural region) hospitals. In this study, 70.2% of tertiary hospitals were located in urban region. In Korea, the mean age of the population in suburban and rural region was higher than that in urban region and comorbidities (hypertension, diabetes, and congestive heart failure) were more prevalent in suburban and rural regions^15^. This difference between urban and rural region were also reported in previous studies^16–18^.

Tertiary hospital is a medical facility which provides a high degree of subspecialty expertise for patients^19^ and is generally larger and provide more procedure compare to non-tertiary hospital. Consequently, tertiary hospital performed more CIED procedure than non-tertiary hospital (62 versus 12 per year) in this study. Several previous studies demonstrated that high procedure volume hospital is less likely to have an adverse outcome after CIED implantation. Nowak et al. reported that hospital annual PPM volume was inversely related to surgical complications and atrial or ventricular dislocation^20^. Similar trends were also observed in ICD^21^. Unlike previous studies, however, we did not observe a better prognosis among patients in the tertiary hospital, high procedure volume hospital, compare to non-tertiary hospital. This might be explained by the difference in the definition of the study outcomes compared to previous studies. Most previous studies defined the study outcomes to include not only death but also traumatic complications (pneumothorax, hemothorax, pericardial effusion, and pericardial tamponade), lead-related complications, and device-related infection^20,21^. When the definition of study outcome was confined to mortality, however, procedure volume did not have a predictive value. Freeman et al. reported that adverse events (including cardiac arrest, cardiac perforation, pneumothorax, hemothorax, lead dislodgement, and device-related infection) were significantly higher in the lowest-procedure volume quartile compared to the highest-procedure volume quartile (OR 1.26, 95% CI 1.05–1.52, *p* < 0.0001)^21^. However, in-hospital death was not different according to procedure volume. Another study also did not show a consistent procedure volume-mortality relationship^22^. In addition to procedure volume, tertiary hospitals are teaching hospital and have more medical personnel, facility, and equipment compared to non-tertiary hospitals because designation criteria for tertiary hospitals are consist of these hospital characteristics^11^. However, we did not observe a better prognosis among patients in the tertiary hospital compare to non-tertiary hospital. This is consistent with previous study^22^.

Several previous studies have shown that operator characteristics are associated with clinical outcomes. Al-Khatib et al. showed that although there was no correlation between operator volume of ICD implantation and 90-day mortality, 90-day rates of mechanical complication (OR 1.47, 95% CI 1.09–1.99) and ICD infection (OR 2.47, 95% CI 1.18–5.17) were significantly higher among operator in the lowest volume quartile compared with those in the highest volume quartile^23^. Curtis et al. reported that ICD implantations by a non-electrophysiologist were associated with a higher risk of in-hospital procedural complications compared to ICD implantation by an electrophysiologist^24^. Our study did not capture the risk associated with the operator characteristics. Therefore, further clinical studies are needed to evaluate it.

Concentration of patients in tertiary hospitals causes not only the economic problem in terms of waste of health care resource and the loss of opportunity cost for other hospitals but also the delay of timely treatment. Previous studies showed that patients tend to make choices based on hospital size, facility, modernity, and professional credibility^7,8^. The phenomenon of the concentration in tertiary hospitals might derive due to the lack of information (such as clinical outcomes). The results of the present study could be helpful for patients’ decision to choose the hospitals and might alleviate the concentration of patients to tertiary hospitals in CIED procedures.

This study had several limitations. First, this study was a retrospective, observational study. Although we rigorously adjusted for baseline covariates using propensity-score matching, there are inherent limitations of a non-randomized study. Second, our study was based on administrative data from the HIRA in South Korea. In patients with CIED, procedural and device-related complications, such as traumatic-, lead-related complications, lead dislodgement, and device-related infection, are important clinical outcomes in addition to mortality. Similar to previous study using administrative databases^22^, this study could not identify these specific types of complications. In addition, we did not specify the cause of death. However, mortality is the most powerful hard endpoint and unbiased endpoint. Furthermore, to the best of our knowledge, this study is the largest study for mortality in Asian patients undergoing first and new implantation of CIED. Finally, this study only included the Korean population, and this may limit the applicability of our findings to other countries. However, considering the paucity of data concerning Asian populations, we believe that this study may have clinical implications.

In conclusion, this nationwide study suggested that mortality was not different between tertiary and non-tertiary hospitals in Korean patients undergoing first and new implantation of CIED. Further prospective, national cohort studies are needed to confirm these findings.

## Supplementary Information

Supplementary Information.

## Data Availability

The present study analyzed the NHI claims data in South Korea. Data of the NHI claims are accessible to researchers after permission of the HIRA in South Korea. Qualified, interested researchers may request access to these data from the HIRA (http://opendata.hira.or.kr/home.do). The authors do not have any special access privileges to these data.
