# Supplementary material for: Comparison of new implantation of cardiac implantable electronic device between tertiary and non-tertiary hospitals: a Korean nationwide study
Source: Sci Rep. 2021 Mar 15;11:3705. doi: 10.1038/s41598-021-83160-w (PMC7961055; doi:10.1038/s41598-021-83160-w)
Supplement: Supplementary file 1 — Supplementary Information. [file 41598_2021_83160_MOESM1_ESM.docx]

**Comparison of New Implantation of Cardiac Implantable Electronic Device between Tertiary and Non-tertiary Hospitals: A Korean Nationwide Study**

Seungbong Han, PhD^1^, Gyung-Min Park, MD^2^, Yong-Giun Kim, MD^2,*^, Ki Won Hwang, MD^3^, Chang Hee Kwon, MD^4^, Jae-Hyung Roh, MD^5^, Sangwoo Park, MD^2^, Ki-Bum Won, MD^2^, Soe Hee Ann, MD^2^, Shin-Jae Kim, MD^2^, Sang-Gon Lee MD^2^.

^1^Department of Biostatistics, College of Medicine, Korea University, Seoul, Korea.

^2^Department of Cardiology, Ulsan University Hospital, University of Ulsan College of Medicine, Ulsan, Korea.

^3^Division of Cardiology, Pusan National University Yangsan Hospital, Pusan National University of Medicine, Yangsan, Korea.

^4^Division of Cardiology, Department of Internal Medicine, Konkuk University Medical Center, Konkuk University School of Medicine, Seoul, Korea.

^5^Division of Cardiology, Chungnam National University Sejong Hospital, Chungnam National University School of Medicine, Sejong, Korea.

*corresponding author: Yong-Giun Kim, E-mail: apollo0822@naver.com

**Supplementary Table S1.** Characteristics of patients undergoing new implantation of ICD in tertiary and non-tertiary hospital between 2013 and 2017 according to subtypes of ICD.

|  | **Single/dual chamber ICD (n=3,043)** | | | **CRT-D (n=721)** | | |
| --- | --- | --- | --- | --- | --- | --- |
| **Characteristics** | **Tertiary Hospital** | **Non-tertiary Hospital** | ***p* value** | **Tertiary Hospital** | **Non-tertiary Hospital** | ***p* value** |
|  | **(n=2,240)** | **(n=803)** |  | **(n=528)** | **(n=193)** |  |
| Enrolled number (%) |  |  | 0.12 |  |  | 0.04 |
| July 2013 to June 2014 | 434 (19.4%) | 146 (18.2%) |  | 119 (22.5%) | 34 (17.6%) |  |
| July 2014 to June 2015 | 567 (25.3%) | 175 (21.8%) |  | 129 (24.4%) | 38 (19.7%) |  |
| July 2015 to June 2016 | 590 (26.3%) | 230 (28.6%) |  | 139 (26.3%) | 49 (25.4%) |  |
| July 2016 to June 2017 | 649 (29.0%) | 252 (31.4%) |  | 141 (26.7%) | 72 (37.3%) |  |
| Age, years | 55.0±15.4 | 58.6±14.8 | <0.001 | 65.6±11.5 | 68.6±10.0 | 0.001 |
| CIED rate by age category |  |  | <0.001 |  |  | 0.15 |
| < 20 | 35 (1.6%) | 3 (0.4%) |  | 2 (0.4%) | 0 (0.0%) |  |
| 20-60 | 1,331 (59.4%) | 403 (50.2%) |  | 146 (27.7%) | 39 (20.2%) |  |
| 61-80 | 821 (36.7%) | 361 (45.0%) |  | 349 (66.1%) | 139 (72.0%) |  |
| > 80 | 53 (2.4%) | 36 (4.5%) |  | 31 (5.9%) | 15 (7.8%) |  |
| Male (%) | 1,682 (75.1%) | 618 (77.0%) | 0.32 | 308 (58.3%) | 107 (55.4%) | 0.50 |
| Comorbid conditions (%) |  |  |  |  |  |  |
| Hypertension | 1,478 (66.0%) | 569 (70.9%) | 0.01 | 490 (92.8%) | 184 (95.3%) | 0.31 |
| Diabetes | 720 (32.1%) | 306 (38.1%) | 0.002 | 261 (49.4%) | 99 (51.3%) | 0.68 |
| Diabetes with chronic complications^*^ | 0 (0.0%) | 1 (0.1%) | 0.26 | 2 (0.4%) | 1 (0.5%) | >0.99 |
| Dyslipidemia | 1,218 (54.4%) | 493 (61.4%) | 0.001 | 367 (69.5%) | 153 (79.3%) | 0.01 |
| Congestive heart failure | 1,038 (46.3%) | 431 (53.7%) | <0.001 | 483 (91.5%) | 168 (87.0%) | 0.09 |
| Peripheral vascular disease | 163 (7.3%) | 60 (7.5%) | 0.88 | 58 (11.0%) | 17 (8.8%) | 0.49 |
| Cerebrovascular disease | 259 (11.6%) | 105 (13.1%) | 0.26 | 60 (11.4%) | 26 (13.5%) | 0.44 |
| Chronic pulmonary disease | 390 (17.4%) | 163 (20.3%) | 0.07 | 148 (28.0%) | 62 (32.1%) | 0.31 |
| Moderate to severe liver disease | 0 (0.0%) | 0 (0.0%) | N/A | 0 (0.0%) | 0 (0.0%) | N/A |
| Renal disease | 173 (7.7%) | 65 (8.1%) | 0.76 | 86 (16.3%) | 29 (15.0%) | 0.73 |
| Cancer | 46 (2.1%) | 17 (2.1%) | 0.89 | 11 (2.1%) | 5 (2.6%) | 0.78 |
| Rheumatic disease | 8 (0.4%) | 0 (0.0%) | 0.12 | 4 (0.8%) | 2 (1.0%) | 0.66 |
| Atrial fibrillation | 484 (21.6%) | 176 (21.9%) | 0.88 | 94 (17.8%) | 29 (15.0%) | 0.43 |
| Ventricular tachyarrhythmia | 488 (21.8%) | 130 (16.2%) | 0.001 | 49 (9.3%) | 15 (7.8%) | 0.66 |
| Aborted cardiac arrest | 434 (19.4%) | 111 (13.8%) | <0.001 | 12 (2.3%) | 2 (1.0%) | 0.37 |
| Charlson comorbidity index | 1.70±1.58 | 1.98±1.77 | <0.001 | 2.70±1.54 | 2.73±1.65 | 0.89 |
| Type of ICD |  |  | <0.001 |  |  |  |
| Single chamber | 1,305 (58.3%) | 527 (65.6%) |  | - | - |  |
| Dual chamber | 935 (41.7%) | 276 (34.4%) |  | - | - |  |
| CRT–D | - | - |  | 528 (100.0%) | 193 (100.0%) |  |
| Indication of ICD |  |  | <0.001 |  |  | 0.28 |
| Primary prevention | 624 (27.9%) | 280 (34.9%) |  | 449 (85.0%) | 171 (88.6%) |  |
| Secondary prevention | 1,616 (72.1%) | 523 (65.1%) |  | 79 (15.0%) | 22 (11.4%) |  |
| In-hospital mortality | 10 (0.4%) | 3 (0.4%) | 0.89 | 6 (1.1%) | 1 (0.5%) | 0.68 |

Data are reported as mean ± SD or as number (%).

CRT–D = cardiac resynchronization therapy with defibrillator; ICD = implantable cardioverter-defibrillator.

^*^including diabetic nephropathy, retinopathy, or neuropathy.

**Supplementary Table S2.** Baseline characteristics of the propensity-score matched patients undergoing new implantation of ICD in tertiary and non-tertiary hospital according to subtypes of ICD.

|  | **Single/dual chamber ICD (n=799 pairs)** | | | **CRT-D (n=191 pairs)** | | |
| --- | --- | --- | --- | --- | --- | --- |
| **Characteristics** | **Tertiary Hospital** | **Non-tertiary Hospital** | ***p* value** | **Tertiary Hospital** | **Non-tertiary Hospital** | ***p* value** |
|  | **(n=799)** | **(n=799)** |  | **(n=191)** | **(n=191)** |  |
| Age, years | 57.9±14.9 | 58.6±14.8 | 0.94 | 67.3±10.0 | 68.4±10.0 | 0.51 |
| Male (%) | 597 (74.7%) | 615 (77.0%) | 0.95 | 114 (59.7%) | 106 (55.5%) | 0.20 |
| Comorbid conditions (%) |  |  |  |  |  |  |
| Hypertension | 584 (73.1%) | 566 (70.8%) | 0.60 | 186 (97.4%) | 182 (95.3%) | 0.18 |
| Diabetes | 296 (37.0%) | 303 (37.9%) | 0.31 | 93 (48.7%) | 99 (51.8%) | 0.61 |
| Diabetes with chronic complications^*^ | 0 (0.0%) | 0 (0.0%) | N/A | 2 (1.0%) | 1 (0.5%) | >0.99 |
| Dyslipidemia | 496 (62.1%) | 490 (61.3%) | 0.69 | 152 (79.6%) | 151 (79.1%) | 0.36 |
| Congestive heart failure | 435 (54.4%) | 428 (53.6%) | 0.74 | 169 (88.5%) | 167 (87.4%) | 0.61 |
| Peripheral vascular disease | 55 (6.9%) | 59 (7.4%) | >0.99 | 14 (7.3%) | 17 (8.9%) | 0.70 |
| Cerebrovascular disease | 106 (13.3%) | 105 (13.1%) | 0.55 | 32 (16.8%) | 25 (13.1%) | 0.58 |
| Chronic pulmonary disease | 174 (21.8%) | 163 (20.4%) | 0.27 | 49 (25.7%) | 61 (31.9%) | 0.40 |
| Moderate to severe liver disease | 0 (0.0%) | 0 (0.0%) | N/A | 0 (0.0%) | 0 (0.0%) | N/A |
| Renal disease | 63 (7.9%) | 65 (8.1%) | 0.50 | 28 (14.7%) | 29 (15.2%) | 0.58 |
| Cancer | 11 (1.4%) | 14 (1.8%) | >0.99 | 4 (2.1%) | 5 (2.6%) | 0.18 |
| Rheumatic disease | 0 (0.0%) | 0 (0.0%) | N/A | 2 (1.0%) | 2 (1.0%) | 0.62 |
| Atrial fibrillation | 166 (20.8%) | 175 (21.9%) | 0.53 | 28 (14.7%) | 29 (15.2%) | 0.78 |
| Ventricular tachyarrhythmia | 118 (14.8%) | 129 (16.1%) | 0.17 | 17 (8.9%) | 15 (7.9%) | 0.35 |
| Aborted cardiac arrest | 98 (12.3%) | 110 (13.8%) | 0.93 | 3 (1.6%) | 2 (1.0%) | >0.99 |
| Charlson comorbidity index | 1.95±1.64 | 1.95±1.73 | 0.27 | 2.63±1.61 | 2.73±1.66 | 0.82 |
| Type of ICD |  |  | 0.46 |  |  |  |
| Single chamber | 509 (63.7%) | 525 (65.7%) |  | - | - |  |
| Dual chamber | 290 (36.3%) | 274 (34.3%) |  | - | - |  |
| CRT–D | - | - |  | 191 (100.0%) | 191 (100.0%) |  |
| Indication of ICD |  |  | 0.86 |  |  | 0.44 |
| Primary prevention | 282 (35.3%) | 278 (34.8%) |  | 167 (87.4%) | 169 (88.5%) |  |
| Secondary prevention | 517 (64.7%) | 521 (65.2%) |  | 24 (12.6%) | 22 (11.5%) |  |

Data are reported as mean ± SD or as number (%).

CRT–D = cardiac resynchronization therapy with defibrillator; ICD = implantable cardioverter-defibrillator.

^*^including diabetic nephropathy, retinopathy, or neuropathy.

**Supplementary Table S3.** Propensity score matched in-hospital mortality and all-cause death of patients undergoing new implantation of ICD in tertiary and non-tertiary hospital according to subtypes of ICD.

| **Propensity-Score Matched Analysis** | **Single/dual chamber ICD**  **(n=799 pairs)** | | **CRT-D**  **(n=191 pairs)** | |
| --- | --- | --- | --- | --- |
|  | Tertiary hospital compared to non-tertiary hospital | | | |
|  | Odds ratio (95% CI) | *p* value | Odds ratio (95% CI) | *p* value |
| **In-hospital mortality** | 0.67 (0.11-3.99) | 0.66 | 2.01 (0.18-22.36) | 0.57 |
|  | Hazard ratio (95% CI) | *p* value | Hazard ratio (95% CI) | *p* value |
| **All-cause Death** | 0.96 (0.70-1.33) | 0.82 | 0.90 (0.55-1.47) | 0.67 |

CI = confidence interval; CRT–D = cardiac resynchronization therapy with defibrillator; ICD = implantable cardioverter-defibrillator.
